# Unsuspected somatic mosaicism for *FBN1* gene contributes to Marfan syndrome

**DOI:** 10.1038/s41436-020-01078-6

**Published:** 2021-01-25

**Authors:** Pauline Arnaud, Hélène Morel, Olivier Milleron, Laurent Gouya, Christine Francannet, Antoine Da Costa, Carine Le Goff, Guillaume Jondeau, Catherine Boileau, Nadine Hanna

**Affiliations:** 1 Université de Paris, Laboratory for Vascular Translational Science, INSERM U1148, Hôpital Bichat-Claude-Bernard, Paris, France; 2 Département de Génétique, Assistance Publique–Hôpitaux de Paris, Hôpital Bichat, Paris, France; 3 Centre national de référence pour le syndrome de Marfan et pathologies apparentés, Assistance Publique–Hôpitaux de Paris, Hôpital Bichat, Paris, France; 4 Service de Génétique Moléculaire Neuro-Vasculaire, Assistance Publique–Hôpitaux de Paris, Hôpital Saint Louis, Paris, France; 5 Service de Génétique Médicale, Pôle Femme et Enfant, Centre Hospitalier Universitaire de Clermont-Ferrand, Hôpital d’Estaing, Clermont-Ferrand, France; 6 Service de Cardiologie, Centre Hospitalier Universitaire de Saint-Etienne, Hôpital Nord, Saint-Etienne, France

## Abstract

**Purpose:**

Individuals with mosaic pathogenic variants in the *FBN1* gene are mainly described in the course of familial screening. In the literature, almost all these mosaic individuals are asymptomatic. In this study, we report the experience of our team on more than 5,000 Marfan syndrome (MFS) probands.

**Methods:**

Next-generation sequencing (NGS) capture technology allowed us to identify five cases of MFS probands who harbored a mosaic pathogenic variant in the *FBN1* gene.

**Results:**

These five sporadic mosaic probands displayed classical features usually seen in Marfan syndrome. Combined with the results of the literature, these rare findings concerned both single-nucleotide variants and copy-number variations.

**Conclusion:**

This underestimated finding should not be overlooked in the molecular diagnosis of MFS patients and warrants an adaptation of the parameters used in bioinformatics analyses. The five present cases of symptomatic MFS probands harboring a mosaic *FBN1* pathogenic variant reinforce the fact that apparently asymptomatic mosaic parents should have a complete clinical examination and a regular cardiovascular follow-up. We advise that individuals with a typical MFS for whom no single-nucleotide pathogenic variant or exon deletion/duplication was identified should be tested by NGS capture panel with an adapted variant calling analysis.

## INTRODUCTION

Marfan syndrome (MIM 154700, MFS) is a hereditary connective tissue disorder with an estimated incidence of 1 in 5,000 individuals. In this disease, many systems are affected with great phenotypic variability and life-threatening complications, such as the cardiovascular system, with thoracic aortic aneurysms and dissections; ocular system, with ectopia lentis; and skeletal system, with recognizable features such as scoliosis, long bone overgrowth, arachnodactyly, and pectus deformity. The clinical diagnosis is based on the revised Ghent nosology.^1^ Heterozygous pathogenic variants in the *FBN1* gene, encoding fibrillin-1, an extracellular matrix protein, are found in the majority of patients with MFS (1,850 different pathogenic variants described in the UMD-FBN1 database^2^ (http://www.umd.be/FBN1/). A clear family history is apparent in the majority of MFS probands, whereas the disease arises de novo in about 25% of the cases.

Mosaicism defines an individual who has developed from a single fertilized egg and has two or more populations of cells with distinct genotypes, due to postzygotic de novo variants.^3^ A variant can occur in a somatic cell and be contained in only a few tissues (somatic mosaicism), it can occur in a germline cell (germline/gonadal mosaicism), or it can occur in an early precursor cell giving a mixed somatic and germline mosaicism (gonosomal mosaicism).^4^ A transmission of the variation to descendants is possible if the mosaic is present in a germ cell, which is the case in germline and gonosomal mosaicism. The most obvious example of somatic mosaicism is cancer, but mosaicism has also been described extensively in autosomal dominant diseases. The phenomenon was initially highlighted through the observation of localized or segmental forms of cutaneous diseases, such as neurofibromatosis type 1.^5^ Another example is Proteus syndrome, which is caused by somatic mosaicism for a pathogenic variant presumed lethal in the nonmosaic state.^6,7^

**Fig. 1:**
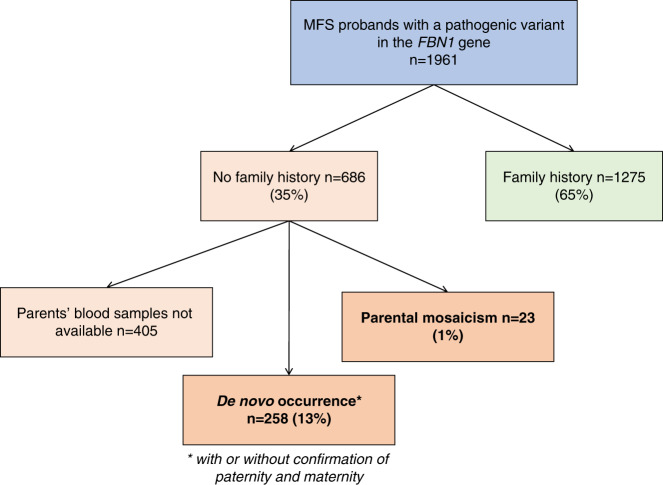
Distribution of sporadic and familial cases in all the Marfan syndrome (MFS) patients with *FBN1* pathogenic variants detected in the laboratory. Percentages are expressed relative to the total number of probands.

Mosaicism was long suspected to exist in MFS because of the high rate of sporadic cases.^8^ Our team performs systematic diagnostic study of the *FBN1* gene in patients suspected for MFS since the early 1990s. More than 5,000 MFS-suspected probands have been tested for molecular diagnosis, either by Sanger sequencing or by next-generation sequencing (NGS) capture panel, and 1,961 were shown to harbor at least one heterozygous pathogenic variant in the *FBN1* gene.^9^ Among these MFS patients with a pathogenic variant in the *FBN1* gene, a family history was documented in 65%, while among the remaining apparently sporadic cases, a de novo occurrence could be confirmed in 258 cases, representing 13% of all the patients (Fig. 1). Moreover, a parental mosaicism was found in 23 cases, notably the first case of both somatic and gonosomal mosaicism reported in MFS.^10^ At the clinical level, all 23 cases were asymptomatic. In the literature, only two cases of symptomatic MFS patients exhibit a *FBN1* mosaic pathogenic variant.^11,12^ The past 20 years have completely altered the diagnosis of MFS and related disorders, first through the availability of capillary Sanger sequencers followed by that of the NGS technologies.^13^ With the latter applied to a capture panel, we discovered five cases of mosaicism in five probands for whom the diagnosis of MFS had already been ascertained at the clinical level. This study reports on these unusual and underestimated molecular events that should be routinely looked for.

## MATERIALS AND METHODS

### Patients

All the patients included were followed either by the Centre National Maladies Rares—Syndrome de Marfan et apparentés, the French National Reference Centre in Paris, or by an affiliated regional Center of Expertise. Clinical diagnosis was established according to the revised Ghent nosology.^1^ Patients were examined by several physicians: cardiologists, ophthalmologists, geneticists, rheumatologists, or pediatricians (depending on their age) with specific evaluation of clinical features included in MFS. Systematic slit-lamp examination, cardiac ultrasonography and radiological investigations were also performed. Aortic diameter was evaluated at the root and at the tubular portion of the ascending aorta at end diastole. Aortic aneurysm was defined as a measure above mean + 2 standard deviations (*Z* score >2) as described by Campens et al.^14^ Dural ectasia was looked for by imaging. Systemic score was calculated as described in the revised Ghent nosology.^1^ Between 1996 and 2020, blood samples were obtained for more than 5,000 consecutive unselected probands referred nationwide to our laboratory for molecular diagnosis of suspected MFS.

### DNA amplification and variant detection

Genomic DNA was isolated from peripheral blood leukocytes with a DNA Blood 4K kit (Perkin Elmer®) on Chemagicstar (Hamilton®) according to the manufacturer’s instructions. Originally, the *FBN1* gene was systematically screened in patients suspected of MFS by bidirectional Sanger sequencing as previously reported.^9^ Since 2014, the *FBN1* gene has been screened on MiSeq (Illumina®) by NGS (on more than 3,000 patients) using MARFAN MASTR Assay (Multiplicom®) or a custom capture array (NimbleGen, Roche®) designed to capture *FBN1* gene (NM_000138.4; genome build hg19) and 27 other genes already known to be associated with Marfan syndrome and related diseases (total size of the target: 132 kb). Variant calling is performed through CLC Genomics Workbench v10.1.1 (Qiagen® Bioinformatics). Once a single-nucleotide or a small insertion/deletion pathogenic variant is found in this way, it is systematically confirmed by bidirectional Sanger sequencing of the altered exon. When the pathogenic variant alters the regional restriction map, the presence of the variation is also checked by polymerase chain reaction (PCR)/digestion using the appropriate restriction enzyme. When possible, familial segregation of pathogenic variants is investigated. Description of sequence variants is performed according to Human Genome Variation Society nomenclature.^15^ In brief, complementary DNA (cDNA) numbering with +1 corresponds to the A of ATG, the translation initiation codon in the reference sequence (*FBN1*: NM_000138.4). Exon numbering is historically made considering that exon 1 carries the initiation codon.

### Copy-number variation (CNV) analysis

Before the routine use of the NimbleGen custom capture array, CNVs in the *FBN1* gene were searched for with multiplex ligation-dependent probe amplification (MLPA, MRC-Holland®) on 53 of the 65 exons of *FBN1* using a ABI-3130XL analyzer, and analyzed using Coffalyser.Net (MRC-Holland®). For patients screened using the NimbleGen custom capture array, CNV analysis was based on a comparison of normalized coverage depths for each amplicon to those of a group of 24 patients from the same experiment (CNV ratio). All CNVs were confirmed by quantitative PCR using specific PCR primers and SYBR™ Green Master Mix (Applied Biosystem®) on an ABI 7500 Fast and analyzed with the 7500 Fast Real-Time PCR System software (Applied Biosystem®). Quantification was normalized using the expression of the two housekeeping genes (*PBGD* and *RB1*).

### Long-range PCR

A 7-kb fragment comprising *FBN1* exons 44 to 50 was amplified using an Expand® High Fidelity PCR System (Sigma-Aldrich®) on a PeqSTAR® thermal cycler (UNO96G), with the following program: initial denaturation: 2 minutes at 94 °C; 10 cycles (denaturation: 10 seconds at 94 °C, annealing: 30 seconds at 60 °C, elongation: 15 min at 68 °C); 25 cycles (denaturation: 15 seconds at 94 °C, annealing: 30 seconds at 60 °C, elongation: 15 minutes + 20 seconds at 68 °C); final elongation: 7 minutes at 68 °C. Multiple amplification primers were designed in introns 44 and 49, and sequentially used to amplify shorter and shorter fragments. The shortest amplified fragment was then sequenced and analyzed using Seqscape® (Applied Biosystem®) to identify breakpoints in these introns.

## RESULTS

### Molecular aspects

**Fig. 2:**
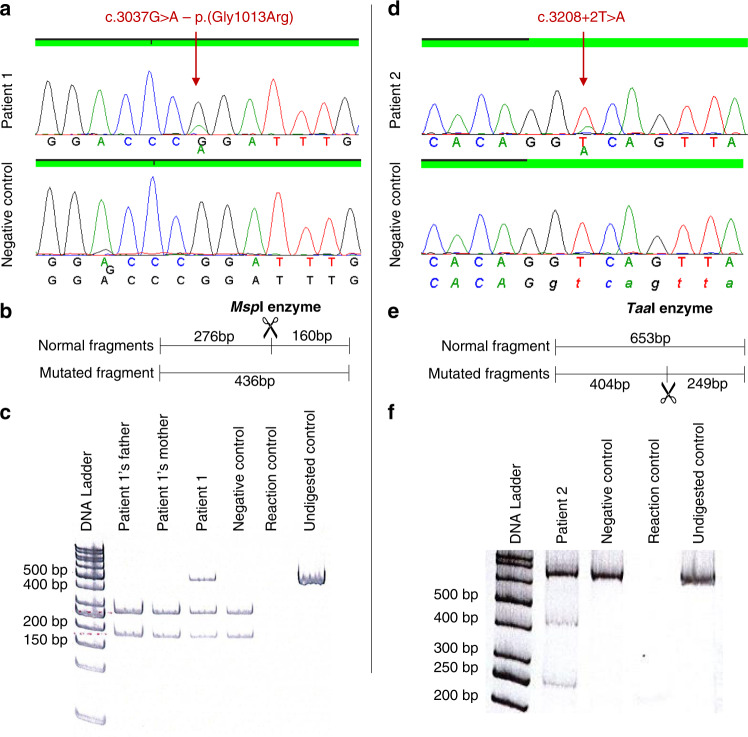
Results of the mosaic pathogenic variant confirmations for patient 1 and 2. Targeted Sanger sequencing on exon 24 (patient 1, **a**) and exon 25 (patient 2, **d**). Similar sequencing profiles were obtained for patient 3 and 4. Restriction maps and results of the enzymatic restriction using either *Msp*I enzyme (patient 1, **b**,**c**) or *Taa*I enzyme (patient 2, **e**,**f**).

**Fig. 3:**
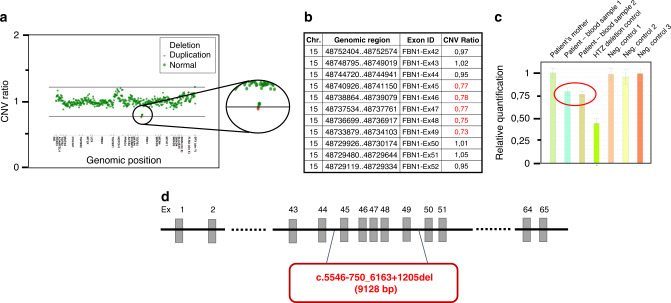
Characterization of the *FBN1* mosaic exons deletion identified in patient 5. (**a**) Scatter plot of copy-number variation (CNV) for the next-generation sequencing (NGS) capture panel (28 genes). (**b**) CNV ratio data from NGS for exons 42 to 52 in the *FBN1* gene. (**c**) Relative quantification of exon 49 (samples from left to right: patient’s mother, patient blood sample 1, patient blood sample 2, heterozygous deletion control, negative controls 1, 2, and 3). Similar profiles were obtained for exons 45 and 47. (**d**) Schematic view of the deletion breakpoints in the *FBN1* gene in this proband.

Of 5,000 MFS suspected patients, 1,961 probands were shown to harbor at least one heterozygous pathogenic variant in the *FBN1* gene.^9^ Approximately half of these were identified by NGS capture panel routinely performed since 2014. This last technology allowed the identification of five mosaic pathogenic variants in the *FBN1* gene in five different probands. In these five cases, the entire *FBN1* gene was covered with a minimum depth of 90 reads (mean depth: 422, 453, 478, 235, and 927 respectively for patients 1 to 5). In four cases (patients 1 to 4), four different single-nucleotide variants were identified with abnormally low allelic fraction (26%, 18%, 20%, and 24% respectively). The presence of these mosaic variants was confirmed using targeted Sanger sequencing, and PCR followed by enzymatic restriction when appropriate (Fig. 2). The variant found in patient 1 (c.3037G>A–p.[Gly1013Arg]) affects a highly conserved glycine, consensus in the transforming growth factor-β binding protein domain 3, and has already been reported in ten probands in UMD-FBN1 database. Prediction tools were in favor of a pathogenic effect for this variation (UMD-Predictor®,^16^ PolyPhen-2^17^). In patient 2, an intronic variant was detected in intron 25 (c.3208+2T>A). This variant affects the consensus donor splice site of intron 25 and is predicted to cause an abnormal splicing of the intron and a major impact at the protein level. The two mosaic variants identified in patients 3 and 4 affect a cysteine, in calcium-binding epidermal growth factor domains 12 and 19, respectively. These two pathogenic variants, already reported in UMD-FBN1 database, are known to affect proper disulfide bond formation and to disrupt domain conformation. In the case of patient 5, a mosaic deletion of exons 45 to 49 in the *FBN1* gene was suspected during CNV analysis since CNV ratios were intermediate (ranging from 0.73 to 0.78) for these five exons (Fig. 3). Quantitative PCR confirmed a 75–80% relative quantity of exons 45, 47, and 49, compared with controls. Long-range PCR was performed to determine the deletion breakpoints and identified a 9,128-bp deletion (c.5546-750_6163 + 1205del). This deletion of five exons is predicted to lead to an in-frame deletion of 206 amino acids but this could not be verified by a transcript analysis.

### Clinical aspects

The five probands with a mosaic pathogenic variant described in this report were sporadic cases. None of them had children, and their unaffected parents did not carry the pathogenic variant when the samples were available. Four of five were diagnosed during infancy and displayed classical MFS features usually found in heterozygote patients, with ascending aortic dilatation and/or ectopia lentis, and a systemic score ranging from 6 to 9. The last one (patient 4) was diagnosed after an emergency surgery for type A aortic dissection at 48 years old; the clinical examination was incomplete for him. The detailed clinical features of these probands are summarized in Table 1, as well as with the clinical features of the two symptomatic mosaic patients from the literature.^11,12^

**Table 1:** Clinical features according to the revised Ghent nosology for Marfan syndrome^1^ for the five probands with a *FBN1* mosaic pathogenic variant from this report and for the two symptomatic mosaic patients from the literature.

	Patient 1 (present study)	Patient 2 (present study)	Patient 3 (present study)	Patient 4 (present study)	Patient 5 (present study)	Blyth et al.^11^	Rekondo et al.^12^
***FBN1*** **mosaic pathogenic variant**	c.3037G>A p.(Gly1013Arg)	c.3208+2T>A	c.3221G>A p.(Cys1074Tyr)	c.4139G>A p.(Cys1380Tyr)	c.5546-750_6163+1205del (exons 45-49 deletion)	Exons 13–49 deletion	c.2677+5G>C
**Age at diagnosis**	8	3	6	48	5	3	49
**Age at consultation**	23	53	28	48	26	5	58
**Cardiovascular**							
Ascending aortic dilatation	Y	Y	N	Y	Y	N	NA
Valsalva diameter (*z* score (Campens et al.^14^)	44 mm (+3.8 SD)	48 mm (+3.4 SD)	32 mm (+1 SD)	45 mm (+2.7 SD)	43 mm (+3.1 SD)	NA	NA
Ascending aortic dissection	N	N	N	Y	N	N	N
Aortic surgery	N	N	N	Y	N	N	Y
Age/type/indication of surgery	N	N	N	48/Bentall/type A aortic dissection	N	N	49/percutaneous placement of an endovascular prosthesis/type B aortic dissection
Mitral valve prolapse	N	N	N	N	Y	Y	NA
Ascending aortic dilatation or dissection before 40 years old	N	N	N	N	N	N	N
**Ocular**							
Ectopia lentis	Y	Y	Y	N	Y	Y	N
Age at ectopia lentis diagnosis	8	3	6	N	11	3	NA
Myopia (>3 dioptries)	Y	N	N	N	Y	Y	Y
Flat cornea	Y	Y	Y	N	Y	NA	NA
**Musculoskeletal**							
Pectus carinatum	N	N	N	N	Y	Y	N
Severe pectus excavatum	N	Y	N	N	N	N	Y
Dolichostenomelia = reduced US/LS AND increased arm/height AND no severe scoliosis	N	N	N	NA	N	N	NA
Positive wrist and thumb signs (arachnodactyly)	Y	N	Y	NA	Y	Y	NA
Positive wrist or thumb signs	N	Y	N	NA	N	N	NA
Scoliosis >20° or spondylolisthesis	N	Y	N	Y	N	Y	Y
Limited elbow extension <170°	Y	Y	Y	NA	N	NA	NA
Joint hypermobility	N	N	N	NA	Y	Y	NA
Protrusio acetabulae	N	N	N	N	Y	NA	NA
Hindfoot deformity	N	N	N	N	N	NA	NA
Plain pes planus	N	Y	Y	N	N	Y	NA
Typical facial appearance (3/5: dolichocephaly, enophthalmos, downslanting palpebral fissures, malar hypoplasia, retrognathia)	N	Dolichocephaly, downslanting palpebral fissures, malar hypoplasia	N	N	N	Dolichocephaly, malar hypoplasia	NA
Highly arched palate with crowding	N	Y	Y	Y	Y	Y	NA
**Other**							
Pneumothorax	N	N	N	N	N	NA	NA
Striae	Y	N	Y	Y	N	NA	NA
Recurrent herniae	N	N	N	Y	Y	NA	NA
Dural ectasia	Y	Y	N	NA	Y	NA	NA
**Systemic score**	8	7	6	≥2	9	≥8	≥3

*Y* yes, *N* no, *NA* not available, *US/LS* upper segment to lower segment.

## DISCUSSION

In the present study, we describe five MFS probands harboring mosaic *FBN1* pathogenic variants. At the clinical level, these mosaic patients displayed typical MFS features with the involvement of the skeletal, cardiovascular, and/or ocular systems. These findings were unexpected since mosaicism is rare in patients with typical MFS in the literature, which almost exclusively reports asymptomatic mosaic parents in the context of familial studies. Indeed, our team reported the first case of paternal mosaicism with both somatic and gonadal mosaicism for a *FBN1* gene variant.^10^ In sporadic cases of MFS, it is important to find out whether the variation occurred de novo or if it has been inherited, to properly assess the risk of recurrence for another child. In our experience and in the literature, individuals harboring mosaic pathogenic variants in the *FBN1* gene are usually asymptomatic.^2,18–23^ As a precaution, a follow-up in cardiology is recommended for these patients. In the literature, mosaicism is a very rare event in MFS patients since only two reports mention symptomatic probands mosaic for a *FBN1* pathogenic variant.^11,12^ The first case is a sporadic case of MFS diagnosed at a young age for whom a mosaic deletion of exons 13 to 49 was reported. The second report is a symptomatic proband’s mother who was shown to harbor the familial intronic variant as a mosaic.

The five present cases of symptomatic probands mosaic for pathogenic variants in the *FBN1* gene reinforce the fact that caution is warranted in the clinical follow-up of parental mosaic individuals. Another interesting point is that somatic mosaicism could also sometimes explain the mild and incomplete form of the disease often seen in the patients referred to MFS clinics for diagnosis.

We have identified an uncommon phenomenon in the molecular diagnosis of MFS patients. Indeed, for some MFS patients, the causal molecular event may have been missed due to lack of knowledge and technical sensitivity. In the literature, the power of MLPA for the detection of mosaicism in blood samples has been shown to be low, since it could not detect duplication mosaics below 40% or deletion mosaics below 30%.^24^ MLPA is therefore not an effective method for the detection of low-grade mosaicisms and its wide use in the last decade could explain why no mosaics for major rearrangements were seen at the time. Interestingly, targeted Sanger sequencing allows detection of mosaic variants with far lower allele fractions, as low as 8% according to a recent study.^25^ However, the conditions in which this percentage was obtained are not applicable to routine screening by Sanger sequencing. In our experience, enzymatic restriction is more sensitive than Sanger sequencing and useful in targeted familial screening to accurately detect parental mosaicism. NGS appears to be the ideal method to detect mosaic variants, provided that background signal is low enough to be to detect mosaic variants. Therefore, each capture method using custom bioinformatics analyses should be adapted to properly query for this molecular phenomenon and should be combined with additional Sanger sequencing. The lowest allele fraction detected in this study was 18%; however, the minimal detectable allele fraction is expected to be lower with an adapted bioinformatics analysis. Of note, we were able to detect a 4% minor allele fraction at another locus in the context of parental mosaicism (unpublished data). Furthermore, parental samples are tested using NGS technology in our laboratory to accurately detect parental mosaicism, as also suggested by Brewer et al.^25^ This is particularly warranted to properly evaluate the risk of recurrence that another child will be heterozygous for the familial *FBN1* pathogenic variant.

In our experience acquired from mosaic parents, the rate of heterozygous cells was identical in buccal swab and blood sample. Unfortunately, we were not able to test any other tissue from the five MFS patients with a *FBN1* mosaic pathogenic variant, but we can assume that in affected tissues, cardiovascular and ophthalmological tissues for instance, the rate of heterozygous cells might be higher. Further studies need to be done on other tissues to evaluate the presence of mosaic pathogenic variants in the *FBN1* gene.

Taken together, the seven mosaic variants detailed in this study and in the literature are three missense variants, two variants with a predicted impact on splicing, and two deletions of several successive exons. Interestingly, three of the seven mosaic variants are located in the “neonatal region” corresponding to exons 24 to 32 of the *FBN1* gene. This region has been associated with more severe phenotypes, including neonatal MFS.^26^ Moreover, the missense pathogenic variant identified in patient 1 was also found in five other probands in our laboratory and all of them displayed a severe form (infantile or neonatal) of the disease. Since patient 1 did not display a particularly severe form of the disease, it could be speculated that a mosaic state could influence the severity of the disease. Furthermore, we can hypothesize that the particularly deleterious consequences of these seven variants may explain why these mosaic patients are symptomatic.

Interestingly, combining the results from this report and from the literature, two mosaic patients of seven concerned a deletion of several exons in the *FBN1* gene. This type of molecular event may be difficult to identify and the threshold for CNV analysis must be set keeping in mind the possibility of mosaicism.

These five pathogenic variants have been identified through NGS capture panel technology with a high mean depth on the entire *FBN1* gene (>90 reads). We hypothesized that some cases of mosaicisms could have been missed while performing screening for *FBN1* variants using Sanger sequencing before 2014. We advise that individuals with a typical MFS for whom no pathogenic single-nucleotide variant or exons deletion/duplication was identified should be tested by NGS capture panel with an adapted variant calling analysis.

### Conclusion

Herein, we report five cases of symptomatic MFS patients with a mosaic *FBN1* pathogenic variant, discovered in the course of molecular diagnosis thanks to NGS capture panel technology. This uncommon molecular event should not be overlooked and warrants adapting the parameters used in bioinformatics analyses. Retrospectively, it is highly probable that the phenomenon was indeed overlooked and mosaic variations have been missed in MFS probands. This has important consequences since these patients are now likely to be offered costly sequencing of their genome that, in fact, is not necessary. Finally, the five present cases of symptomatic patients with a mosaic *FBN1* pathogenic variant underscores the need to perform complete clinical follow-up in apparently asymptomatic mosaic parents.

## Data Availability

The data are available upon request.

## References

[1] Loeys BL, Dietz HC, Braverman AC (2010). The revised Ghent nosology for the Marfan syndrome. J. Med. Genet..

[2] Collod-Béroud G, Le Bourdelles S, Ades L (2003). Update of the UMD-FBN1 mutation database and creation of an FBN1 polymorphism database. Hum. Mutat..

[3] Biesecker LG, Spinner NB (2013). A genomic view of mosaicism and human disease. Nat. Rev. Genet..

[4] Spinner NB, Conlin LK (2014). Mosaicism and clinical genetics. Am J. Med. Genet. C Semin. Med. Genet..

[5] García-Romero MT, Parkin P, Lara-Corrales I (2016). Mosaic neurofibromatosis type 1: a systematic review. Pediatr. Dermatol..

[6] Biesecker, L. G. & Sapp, J. C. in *GeneReviews* (eds Adam, M. P., Ardinger, H. H. & Pagon, R. A. et al.). Proteus syndrome (University of Washington, Seattle, 1993). http://www.ncbi.nlm.nih.gov/books/NBK99495/22876373

[7] Lindhurst MJ, Sapp JC, Teer JK (2011). A mosaic activating mutation in AKT1 associated with the Proteus syndrome. N. Engl. J. Med..

[8] Verstraeten A, Alaerts M, Van Laer L, Loeys B (2016). Marfan syndrome and related disorders: 25 years of gene discovery. Hum. Mutat..

[9] Arnaud P, Hanna N, Aubart M (2017). Homozygous and compound heterozygous mutations in the FBN1 gene: unexpected findings in molecular diagnosis of Marfan syndrome. J. Med. Genet..

[10] Collod-Béroud G, Lackmy-Port-Lys M, Jondeau G (1999). Demonstration of the recurrence of Marfan-like skeletal and cardiovascular manifestations due to germline mosaicism for an FBN1 mutation. Am. J. Hum. Genet..

[11] Blyth M, Foulds N, Turner C, Bunyan D (2008). Severe Marfan syndrome due to FBN1 exon deletions. Am J. Med. Genet. A.

[12] Rekondo J, Robledo-Inarritu M, Vado Y, Pérez de Nanclares G, Arós F (2016). Marfan syndrome caused by somatic mosaicism in an FBN1 splicing mutation. Rev. Espanola Cardiol. Engl. Ed..

[13] Arnaud P, Hanna N, Benarroch L (2019). Genetic diversity and pathogenic variants as possible predictors of severity in a French sample of nonsyndromic heritable thoracic aortic aneurysms and dissections (nshTAAD). Genet. Med..

[14] Campens L, Demulier L, De Groote K (2014). Reference values for echocardiographic assessment of the diameter of the aortic root and ascending aorta spanning all age categories. Am. J. Cardiol..

[15] den Dunnen JT, Dalgleish R, Maglott DR (2016). HGVS recommendations for the description of sequence variants: 2016 update. Hum. Mutat..

[16] Salgado D, Desvignes J-P, Rai G (2016). UMD-Predictor: a high-throughput sequencing compliant system for pathogenicity prediction of any human cDNA substitution. Hum. Mutat..

[17] Adzhubei IA, Schmidt S, Peshkin L (2010). A method and server for predicting damaging missense mutations. Nat. Methods.

[18] Montgomery RA, Geraghty MT, Bull E (1998). Multiple molecular mechanisms underlying subdiagnostic variants of Marfan syndrome. Am. J. Hum. Genet..

[19] Rantamäki T, Kaitila I, Syvänen AC, Lukka M, Peltonen L (1999). Recurrence of Marfan syndrome as a result of parental germ-line mosaicism for an FBN1 mutation. Am. J. Hum. Genet..

[20] Tekin M, Cengiz FB, Ayberkin E (2007). Familial neonatal Marfan syndrome due to parental mosaicism of a missense mutation in the FBN1 gene. Am J. Med. Genet. A.

[21] Hilhorst-Hofstee Y, Hamel BCJ, Verheij JBGM (2011). The clinical spectrum of complete FBN1 allele deletions. Eur. J. Hum. Genet..

[22] Sípek A, Grodecká L, Baxová A (2014). Novel FBN1 gene mutation and maternal germinal mosaicism as the cause of neonatal form of Marfan syndrome. Am J. Med. Genet. A.

[23] Martínez-Quintana E, Caballero-Sánchez N, Rodríguez-González F, Garay-Sánchez P, Tugores A (2017). Novel Marfan syndrome-associated mutation in the FBN1 gene caused by parental mosaicism and leading to abnormal limb patterning. Mol. Syndromol..

[24] van Veghel-Plandsoen MM, Wouters CH, Kromosoeto JNR, den Ridder-Klünnen MC, Halley DJJ, van den Ouweland AMW (2011). Multiplex ligation-depending probe amplification is not suitable for detection of low-grade mosaicism. Eur. J. Hum. Genet..

[25] Brewer CJ, Gillespie M, Fierro J (2020). The value of parental testing by next-generation sequencing includes the detection of germline mosaicism. J. Mol. Diagn..

[26] Faivre L, Collod-Beroud G, Loeys BL (2007). Effect of mutation type and location on clinical outcome in 1,013 probands with Marfan syndrome or related phenotypes and FBN1 mutations: an international study. Am. J. Hum. Genet..

